# Supplementary figures and images for: rab-27 acts in an intestinal pathway to inhibit axon regeneration in C. elegans
Source: PLoS Genet. 2021 Nov 24;17(11):e1009877. doi: 10.1371/journal.pgen.1009877 (PMC8612575; doi:10.1371/journal.pgen.1009877)

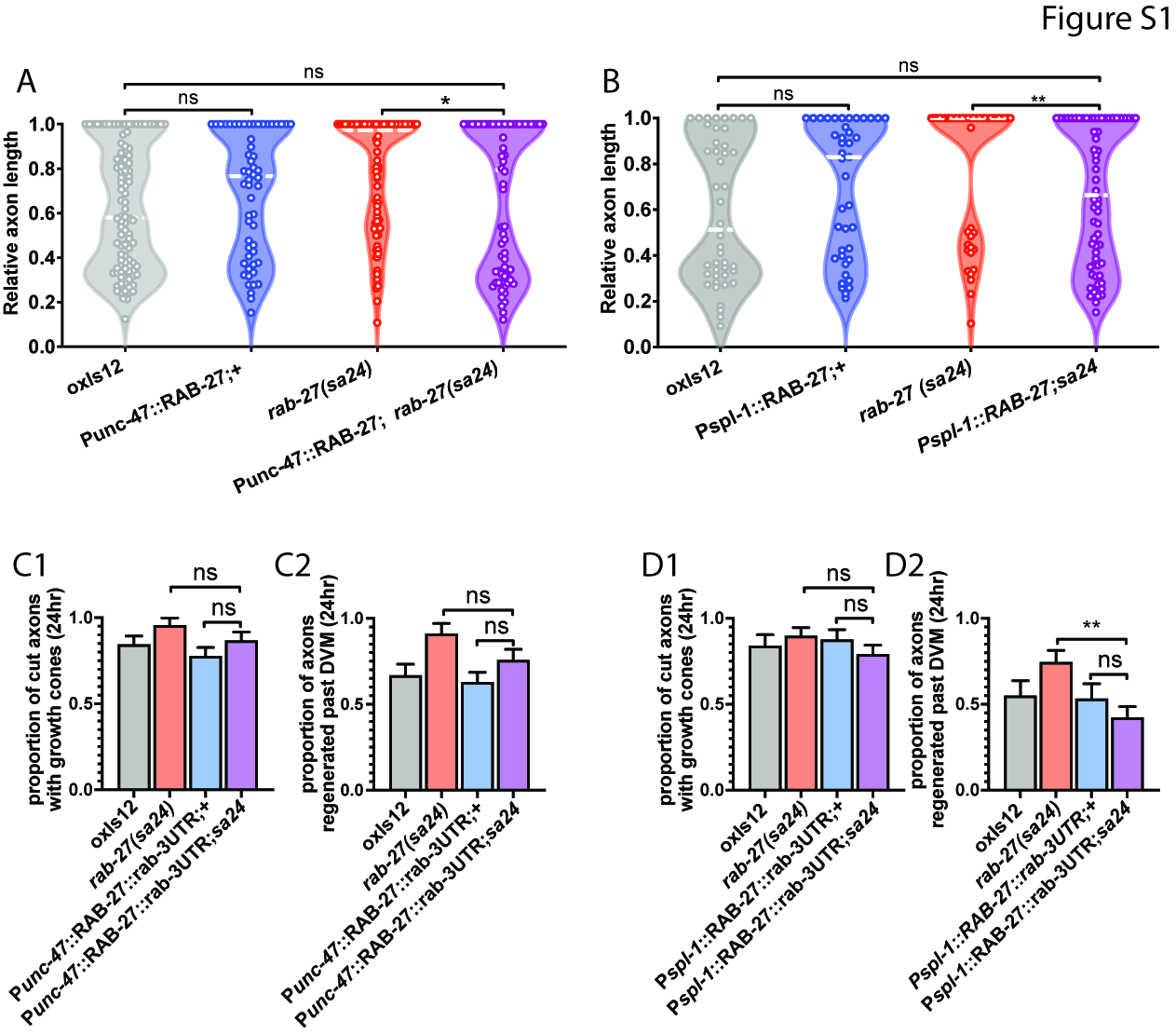

Supplement: S1 Fig — (A-B) Relative axon length in animals expressing RAB-27 cDNA under a GABA neuron-specific (A) or intestine-specific (B) promoter and with unc-54 3’ UTR sequence, in both control (oxIs12) and rab-27 mutant backgrounds. Number of axons cut per genotype, L to R: 51, 67, 22, 45. Kolmogorov-Smirnov test was used. ns, not significant, * p < 0.05, ** p < 0.005, *** p < 0.0005. (C) Proportion of cut axons showing signs of successful regeneration initiation (C1) or regeneration past the dorsoventral midline (C2) in control (oxIs12) and rab-27(sa24) mutant animals, and animals expressing rab-27 cDNA under a GABA neuron-specific promoter (Punc-47) and the rab-3 3’ UTR sequence, in both control and rab-27 mutant backgrounds. Axons were scored after 24 hours of recovery post-axotomy. Axons cut per genotype, L to R: 51, 22, 67, 45. Unpaired t-test was used. ns, not significant. Error bars represent SEM. (D) Proportion of cut axons showing signs of successful regeneration initiation (D1) or regeneration past the dorsoventral midline (D2) in control (oxIs12) and rab-27(sa24) mutant animals, and animals expressing rab-27 cDNA under an intestine-specific promoter (Pspl-1) and the rab-3 3’ UTR sequence, in both control and rab-27 mutant backgrounds. Axons were scored after 24 hours of recovery post-axotomy. Axons cut per genotype, L to R: 31, 39, 32, 57. Unpaired t-test was used. ns, not significant, ** p < 0.005. Error bars represent SEM. (TIF) [file pgen.1009877.s001.tif]

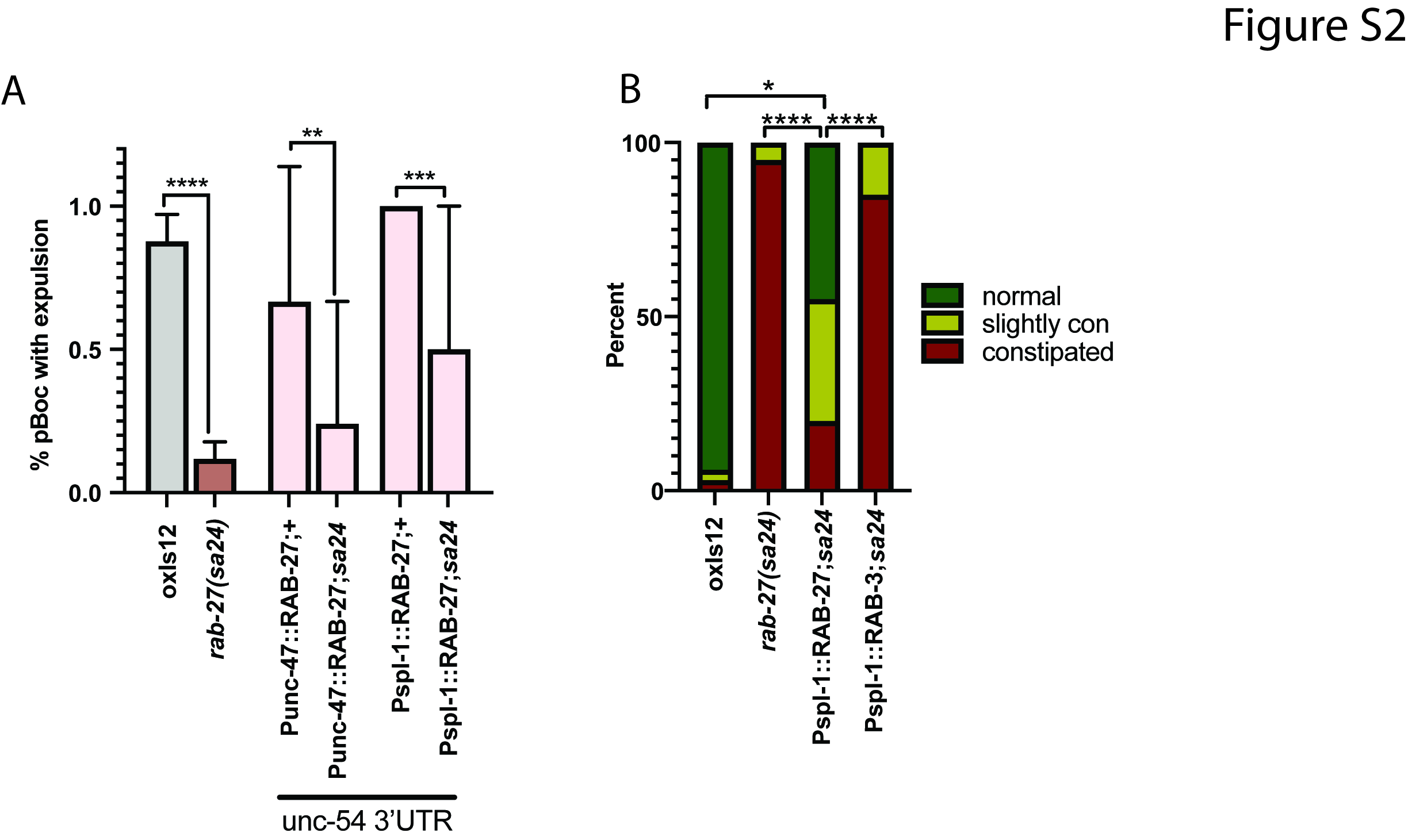

Supplement: S2 Fig — (A) pBoc Re-expression of RAB-27 rescue constructs in the GABAergic neurons were did not rescue DMP defects. Intestinally-expressed RAB-27 cDNA constructs including the unc-54 3’ UTR were not able to restore normal pBoc/exp cycling, unlike constructs containing the rab-3 3’UTR (Fig 2D). pBoc cycles observed, L to R: 49, 119, 27, 25, 20, 18. Kolmogorov-Smirnov test was used. ns, not significant, * p < 0.05, ** p < 0.05, *** p < 0.0005, **** p < 0.0001, Fisher’s Exact Test. Error bars represent SEM. Control: oxIs12. (B) Percent stacked bar graph for visual scoring of Aex phenotype rescue. Animals were randomized on plates and scored by phenotype, then genotyped. Animals were scored as normal (no gut distention, strong pBoc contraction with accompanying expulsion), constipated (severe posterior gut distention, weak pBoc with no expulsion), or slightly con (some possible gut distention, normal pBoc, weak expulsion). Fisher’s Exact test was used. * p < 0.05, **** p < 0.0001. Control: oxIs12. (TIF) [file pgen.1009877.s002.tif]

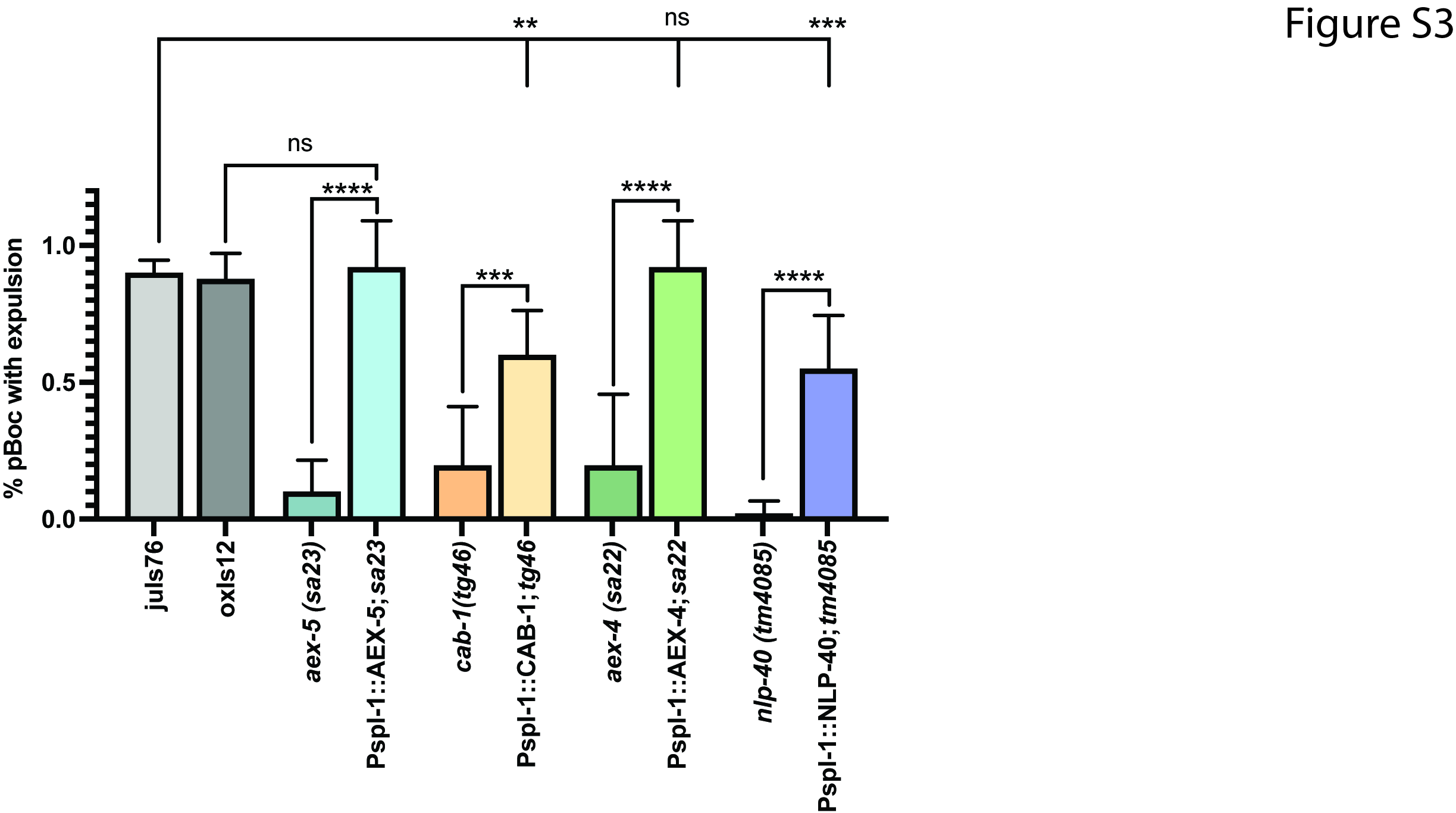

Supplement: S3 Fig — Mutants in the aex pathway that inhibit regeneration also show defects in defecation, caused by a lack of waste expulsion (Exp) following posterior body wall muscle contraction (pBoc). D1 adult animals were randomly selected and observed for 10 DMP cycles, and the ratio of Exp/pBoc was plotted. Intestinal re-expression of aex genes involved in axon regeneration inhibition was sufficient to significantly restore pBoc/Exp ratio in all tested mutants, although Exp/pBoc rescue was not always complete. pBoc cycles observed, L to R: 40, 49, 50, 50, 56, 40, 56, 50, 49. 51. ns, not significant, ** p < 0.05, *** p < 0.0005, **** p < 0.0001, Fisher’s Exact Test. Error bars represent SEM. (TIF) [file pgen.1009877.s003.tif]

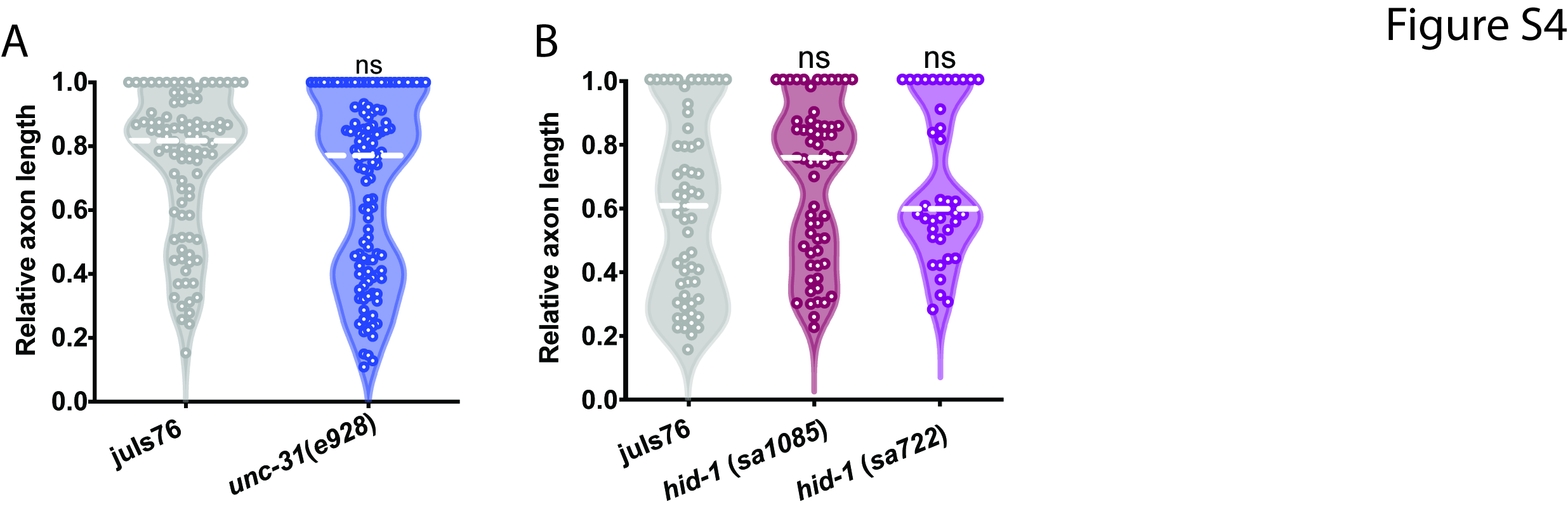

Supplement: S4 Fig — (A) Relative axon length in control (juIs76) and unc-31(e928) mutants. Axons cut per genotype, L to R: 95, 79. Kolmogorov-Smirnov test was used. ns, not significant. (B) Relative axon length in control (juIs76) animals, and hid-1 (js722 and js1058) mutants. Axons cut per genotype, L to R: 57, 61, 40. Kolmogorov-Smirnov test was used. ns, not significant. (TIF) [file pgen.1009877.s004.tif]

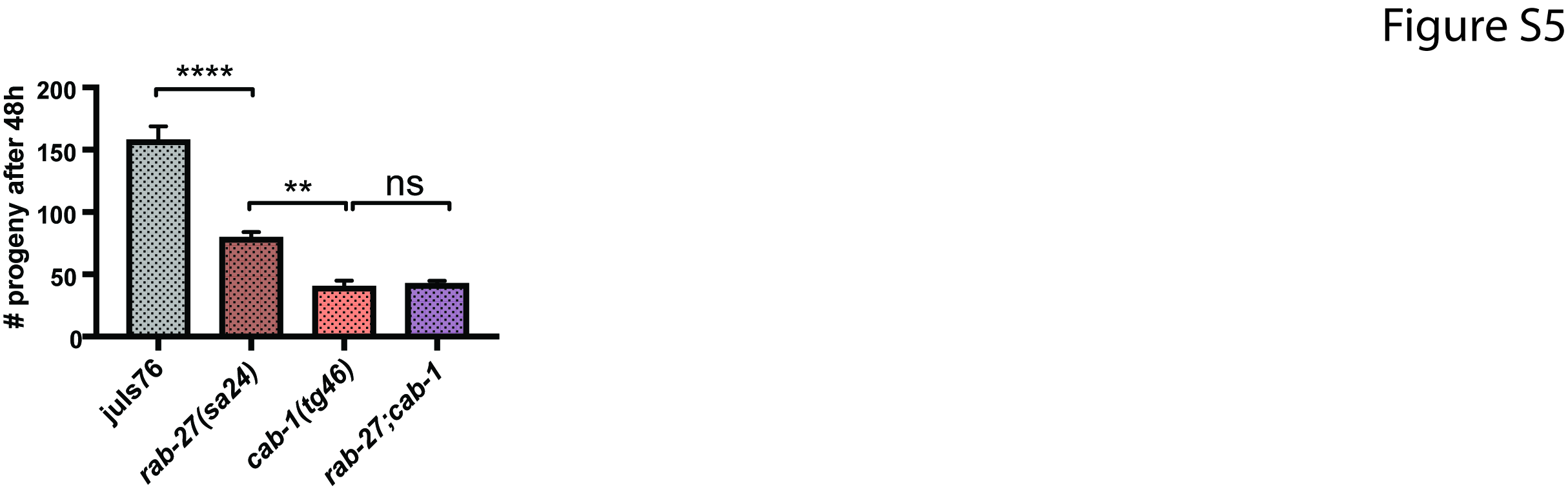

Supplement: S5 Fig — One-day adult worms were placed onto empty NGM plates seeded with OP50 and left for 48 hours. Adults were removed and progeny counted. rab-27 mutants show significantly decreased brood size compared to control animals, and cab-1 mutants show more severe defects. The low brood size of cab-1 mutants is not increased in rab-27;cab-1 double mutants. Worms sampled, L to R: 9, 10, 7, 8. One-way ANOVA test was used. ns, not significant, ** p < 0.005, **** p < 0.0001. Error bars represent SEM. (TIF) [file pgen.1009877.s005.tif]
