# Supplementary material for: rab-27 acts in an intestinal pathway to inhibit axon regeneration in C. elegans
Source: PLoS Genet. 2021 Nov 24;17(11):e1009877. doi: 10.1371/journal.pgen.1009877 (PMC8612575; doi:10.1371/journal.pgen.1009877)
Supplement: S1 Table — (DOCX) [file pgen.1009877.s006.docx]

**Table S1. Generated *C. elegans* strains**

| *rab-27*(*sa24*) I; *oxIs12*[P*unc-47*::GFP;*lin-15+*] X | XE1873 |
| --- | --- |
| wpEx434[P*spl-1*::RAB-27::SL2::mCherry::RAB-3 3’ UTR]; *oxIs12*[P*unc-47*::GFP;*lin-15+*] X | XE2524 |
| wpEx417[P*spl-1*::RAB-27::SL2::mCherry::RAB-3 UTR];*rab-*27(*sa24*) I; *oxIs12*[P*unc-47*::GFP;*lin-15+*] X | XE2452 |
| wpEx418[P*unc-47*::RAB-27::SL2::mCherry::RAB-3 3’ UTR]; *oxIs12*[P*unc-47*::GFP;*lin-15+*] X | XE2451 |
| wpEx436[P*unc-47*::RAB-27::SL2::mCherry::RAB-3 3’ UTR];*rab-27*(*sa24*) I; *oxIs12*[P*unc-47*::GFP;*lin-15+*] X | XE2525 |
| wpEx287[P*unc-47*::RAB-27::SL2::mCherry::UNC-54 3’ UTR]; *oxIs12*[P*unc-47*::GFP;*lin-15+*] X | XE1874 |
| wpEx287[P*unc-47*::RAB-27::SL2::mCherry::UNC-54 3’ UTR];*rab-27*(*sa24*) I; *oxIs12*[P*unc-47*::GFP;*lin-15+*] X | XE1890 |
| wpEx405[P*spl-1*::RAB-27::SL2::mCherry::UNC-54 3’ UTR]; *oxIs12*[P*unc-47*::GFP;*lin-15+*] X | XE2353 |
| wpEx405[P*spl-1*::RAB-27::SL2::mCherry::UNC-54 3’ UTR];*rab-27*(*sa24*) I; *oxIs12*[P*unc-47*::GFP;*lin-15+*] X | XE2354 |
| wpEx288[P*unc-47*::EGFP::RAB-27::UNC-54 3’ UTR]; *wpIs40*[P*unc-47*::*mCherry*] V | XE1904 |
| wpEx435[P*unc-47*::EGFP::RAB-27::UNC-54 3’ UTR; P*unc-47*::mCherry::RAB-3::UNC-54 3’ UTR] | XE2523 |
| *rab-3*(*js49*) II; *oxIs12*[P*unc-47*::GFP;*lin-15+*] X | XE1871 |
| *rbf-1*(*js232*) III; *oxIs12*[P*unc-47*::GFP;*lin-15+*] X | XE1901 |
| *rab-27*(*sa24*) I; *rab-3*(*js49*) II; *oxIs12*[P*unc-47*::GFP;*lin-15+*] X | XE2514 |
| *rab-27*(*sa24*) I; *rbf-1*(*js232*) III; *oxIs12*[P*unc-47*::GFP;*lin-15+*] X | XE2515 |
| wpEx406[P*spl-*1::RAB-3::SL2::mCherry::UNC-54 3’ UTR];*rab-27*(*sa24*) I; *oxIs12*[P*unc-47*::GFP;*lin-15+*] X | XE2351 |
| wpEx406[P*spl-1*::RAB-3::SL2::mCherry::UNC-54 3’ UTR]; *oxIs12*[P*unc-47*::GFP;*lin-15+*] X | XE2352 |
| *aex-1*(*sa9*) I; *oxIs12*[P*unc-47*::GFP;*lin-15+*] X | XE2511 |
| *aex-2*(*sa3*) X; *juIs76*[P*unc-25*::GFP;*lin-15*+] II | XE2517 |
| *aex-3*(*sa5*) X; *juIs76*[P*unc-25*::GFP;*lin-15*+] II | XE2510 |
| *aex-4*(*sa22*) X; *juIs76*[P*unc-25*::GFP;*lin-15*+] II | XE2516 |
| aex-5(sa23) I; *oxIs12*[P*unc-47*::GFP;*lin-15+*] X | XE2509 |
| *unc-31*(*e928*) IV; *oxIs12*[P*unc-47*::GFP;*lin-15+*] X | XE1905 |
| *cab-1*(*tg46*) X; *juIs76*[P*unc-25*::GFP;*lin-15*+] II | XE2512 |
| *cab-1*(*tg46*) X;*rab-27*(*sa24*) I; *juIs76*[P*unc-25*::GFP;*lin-15*+] II | XE2513 |
| *unc-108/rab-2*(*n501*) I; *oxIs12*[P*unc-47*::GFP;*lin-15+*] X | XE2518 |
| *rab-6.2*(*ok2254*) X;*juIs76*[P*unc-25*::GFP;*lin-15*+] II | XE1560 |
| *rab-8*(*tm2526*) I; *oxIs12*[P*unc-47*::GFP;*lin-15+*] X | XE2519 |
| *rab-10*(*q373*) I;*oxIs12*[P*unc-47*::GFP;*lin-15+*] X | XE1804 |
| *rab-18*(*ok2020*) III; *oxIs12*[P*unc-47*::GFP;*lin-15+*] X | XE1872 |
| *rab-19*(*ok1845*) IV; *oxIs12*[P*unc-47*::GFP;*lin-15+*] X | XE2522 |
| *rab-21*(*gk500186*) II; *oxIs12*[P*unc-47*::GFP;*lin-15+*] X | XE2521 |
| *rab-28*(*gk1040*) IV; *oxIs12*[P*unc-47*::GFP;*lin-15+*] X | XE1806 |
| *glo-1*(*zu391*) X; *juIs76*[P*unc-25*::GFP;*lin-15*+] II | XE2520 |
| *nlp-1*(*ok1469*) X; *juIs76*[P*unc-25*::GFP;*lin-15*+] II | XE2409 |
| *nlp-8*(*ok1799*) I; *juIs76*[P*unc-25*::GFP;*lin-15*+] II | XE2407 |
| *nlp-20*(*ok1591*) IV; *juIs76*[P*unc-25*::GFP;*lin-15*+] II | XE2408 |
| *nlp-40(tm4085)* I; *juIs76*[P*unc-25*::GFP;*lin-15*+] II | XE2560 |
| wpEx492[*Pspl-1*::CAB-1::SL2::mCherry::RAB-3 3’UTR]; *cab-1(tg46)* X; *juIs76*[P*unc-25*::GFP;*lin-15*+] II | XE2853 |
| wpEx492[*Pspl-1*::CAB-1::SL2::mCherry::RAB-3 3’UTR]; *juIs76*[P*unc-25*::GFP;*lin-15*+] II | XE2854 |
| wpEx494[*Pspl-1*::AEX-5::SL2::mCherry::RAB-3 3’UTR]; *aex-5(sa23)* I; *oxIs12*[P*unc-47*::GFP;*lin-15+*] X | XE2857 |
| wpEx494[*Pspl-1*::AEX-5::SL2::mCherry::RAB-3 3’UTR]; *oxIs12*[P*unc-47*::GFP;*lin-15+*] X | XE2858 |
| wpEx493[*Pspl-1*::AEX-4::SL2::mCherry::RAB-3 3’UTR]; *aex-4(sa22)* X; *juIs76*[P*unc-25*::GFP;*lin-15*+] II | XE2861 |
| wpEx493[*Pspl-1*::AEX-4::SL2::mCherry::RAB-3 3’UTR]; *juIs76*[P*unc-25*::GFP;*lin-15*+] II | XE2862 |
| wpEx495[*Pspl-1*::NLP-40::SL2::mCherry::RAB-3 3’UTR]; *nlp-40(tm4085)* X*; juIs76*[P*unc-25*::GFP;*lin-15*+] II | XE2864 |
| wpEx495[*Pspl-1*::NLP-40::SL2::mCherry::RAB-3 3’UTR]; *juIs76*[P*unc-25*::GFP;*lin-15*+] II | XE2865 |
| *rab-27*(*sa24*) I; *juIs76*[P*unc-25*::GFP;*lin-15*+] II; *aex-4*(*sa22*) X | XE2867 |
