# Supplementary material for: rab-27 acts in an intestinal pathway to inhibit axon regeneration in C. elegans
Source: PLoS Genet. 2021 Nov 24;17(11):e1009877. doi: 10.1371/journal.pgen.1009877 (PMC8612575; doi:10.1371/journal.pgen.1009877)
Supplement: Attachment — Submitted filename: PLOS Genetics Review rab-27 acts in an intestinal pathway to inhibit axon regeneration in C. elegans..pdf [file pgen.1009877.s007.pdf]

Comments for *rab-27 acts in an intestinal secretory pathway to inhibit axon regeneration in C. elegans*

In their manuscript "*rab-27 acts in an intestinal secretory pathway to inhibit axon regeneration in C. elegans"* the authors show Rab GTPase *rab-27* inhibits GABAergic motor neuron regeneration through activity in the intestine. Overall, the proposed study is interesting and implicates external factors from non-neuronal tissues and cellular factors and long-distance signals to regulate axon regeneration. Most of the conclusions for *rab-27* intestinal dependent pathway is drawn based solely off of loss of function mutant analysis. To fully draw conclusions and implicate a pathway, more genetic analysis needs to be conducted. Below is a list of my comments for the author.

**Major Issues:**

1) Main concern is that most of the genetic pathway arguments are based solely off of whether loss of function in genes involved in DMP signaling or dense core vesicle loading affect GABA neuron axon regeneration. Interestingly loss of function in many of the genes in these pathways do, but there are exceptions *i.e.* genes in these pathways that do not have a phenotype. The way that the manuscript is written, it assumes that *rab-27* is in a pathway with these genes and that the pathway functions similarly to regulate axon regeneration. However, very few follow up experiments are done to show cellular sites of action, evidence to support genetic relationships, or establish a pathway beyond loss of function phenotypes. The author does not show or test genetic doubles that would help determine the genetic interactions to argue that these genes function in common pathways or parallel pathways for axon regeneration. For example, the authors make an argument for NLP-40 and RAB-27 in a dependent pathway, yet they do not show *nlp-40; rab-27* double mutant analysis of axon regeneration. What about specifically, restoring function of *nlp-40* in the intestine in a *rab-27* mutant animal? Similarly, the authors assessed other Rab GTPases and highlight that loss of function in *glo-1* also has an increase in regeneration and is also expressed in the intestine. The authors make an argument that loss of *glo-1* and *rab-27* both have impacts on axon regeneration and are both expressed in the intestine and therefore the intestine plays a role in axon regeneration. However, the authors do not conduct any additional experiments to show a genetic interaction or relationship between *rab-27* and *glo-1*. The authors should make the double mutant *rab-27; glo*-1 to assess the axon regeneration phenotype? Generally, this level of analysis should be done for all of the genes that have phenotypes. You may be working from a hypothesis that implicates DMP signaling pathway, but not all the genes in the pathway have a role in regulating axon regeneration. Therefore, the author needs to show genetic evidence of the genes in the pathway with *rab-27*, similar to what was done with *cab-1*.
2) The tissue-specific experiments raise the question of whether similar or different cellular mechanisms mediate rab-27's regeneration function in the intestine and neurons. The data indicate that *rab-27* can act in either neurons or the intestine to suppress regeneration, but intestinal expression is necessary for complete rescue or restore back to wild type. Have the authors considered co-expressing *rab-27* in both GABA neurons and intestine? This would help the authors determine if *rab-27* has two cellular sites of action and functions in independent parallel pathways perhaps feeding in one a common target.
3) *rab-3* and *rbf-1* do not have roles in axon regeneration, but loss of either can enhance intestinal Rab-27 axon defect. Has the author tested if loss of either *rab-3* and *rbf-1* enhance neuronal Rab-27 axon regeneration phenotype?
4) The author rules out genes like *hid-1* as not having roles in axon regeneration based off no phenotype associated with axon regeneration, but does not generate the double mutant to fully determine if loss of function in *hid-1* would act like *rab-3* or *rbf-1*, either enhance or suppress Rab-27 axon regeneration phenotype.
5) The authors need a working model or schematic to show the potential genetic interactions for *rab-27* and cellular sites of action in GABA axon regeneration. There are at least two independent regulation pathways for inhibition of axon regeneration, a potential pathway mediated by intestinal *rab-27* and another that may be regulated by neuronal *rab-27*.
6) Overall, there are places in the manuscript where there could be more details explaining the rationale for why the authors are testing particular genes or pathways. In addition, there could be more details in the conclusions in explaining the results.

**Minor Comments**

For fecundity assays what was the temperature animals kept?

For statistics and graphing of data, what graphing software was used? This information should be provided.

For the figures, the spacing between figures in panel needs to be adjusted. For example, Figure 1A, is too close to Figure 1C legend. The current layout leads to the reader thinking that the control and rab-27 mutant legend is part of panel A.

In many of the graphs, the white bar is covered or missing, make this stand out more.

For the bar graphs, the individual data points for each animal tested is not provided. For consistency, show the individual data points.
