# Supplementary material for: rab-27 acts in an intestinal pathway to inhibit axon regeneration in C. elegans
Source: PLoS Genet. 2021 Nov 24;17(11):e1009877. doi: 10.1371/journal.pgen.1009877 (PMC8612575; doi:10.1371/journal.pgen.1009877)
Supplement: Attachment — Submitted filename: Reviewer_responses.docx [file pgen.1009877.s008.docx]

**Reviewer Responses:**

**Reviewer 1:**

**Reviewer 1 stated that "** the proposed study is interesting and implicates external factors from non-neuronal tissues and cellular factors and long-distance signals to regulate axon regeneration."

However, the reviewer also stated that "[to] fully draw conclusions and implicate a pathway, more genetic analysis needs to be conducted."

We appreciate the suggestions and have performed additional experiments. We address the specific points in detail below.

1) Main concern is that most of the genetic pathway arguments are based solely off of whether loss of function in genes involved in DMP signaling or dense core vesicle loading affect GABA neuron axon regeneration. Interestingly loss of function in many of the genes in these pathways do, but there are exceptions *i.e.* genes in these pathways that do not have a phenotype. The way that the manuscript is written, it assumes that *rab-27* is in a pathway with these genes and that the pathway functions similarly to regulate axon regeneration. However, very few follow up experiments are done to show **cellular sites of action**, evidence to **support genetic relationships**, or **establish a pathway beyond loss of function phenotypes**. The author does not show or test **genetic doubles** that would help determine the genetic interactions to argue that these genes function in common pathways or parallel pathways for axon regeneration.

**Response**: We appreciate the suggestion to make double mutants. Given the very strong phenotype of *rab-27* mutants, we performed 12 hour regeneration assays to assess *rab-27* double mutants for *cab-1* and *aex-4*, with the idea that this assay would have increased dynamic range. We now show that regeneration in these double mutants is similar to the single mutants. These data are consistent with our proposal that *rab-27, cab-1,* and *aex-4* act in the same pathway to inhibit axon regeneration. These new data are described on p. 3 and presented in Fig. 4C.

However, regeneration is this time point is ongoing (see 10.1016/j.celrep.2014.06.008) making this assay extremely noisy--as can be seen from the distributions in Fig. 4C. Thus, for mutants with a comparatively weak phenotype (such as *nlp-40*), this approach is extremely difficult to power adequately. In other words, it would be very difficult to be sure about whether or not loss of *nlp-40* increases the already high regeneration phenotype of *rab-27* mutants.

As an alternative, we performed additional tissue-specific experiments. We now show that for all identified regeneration inhibitors (*nlp-40*, *cab-1*/NPDC1, *aex-4*/SNAP25, and *aex-5*/KPC3), rescue in the intestine is sufficient to restore axon regeneration to normal levels. These experiments confirm the central idea of the manuscript--that components of a well-characterized intestinal secretory pathway act in that tissue to inhibit axon regeneration. The new data are described on pages 3, 4 and 5 and shown in Figures 3B, 4B and 5B.

 2)	The tissue-specific experiments raise the question of whether similar or different cellular mechanisms mediate rab-27’s regeneration function in the intestine and neurons. The data indicate that *rab-27* can act in either neurons or the intestine to suppress regeneration, but intestinal expression is necessary for complete rescue or restore back to wild type. Have the authors considered **co-expressing *rab-27* in both GABA neurons and intestine**? This would help the authors determine if *rab-27* has two cellular sites of action and functions in independent parallel pathways perhaps feeding in one a common target. *rab-3* and *rbf-1* do not have roles in axon regeneration, but loss of either can enhance intestinal Rab-27 axon defect. Has the author tested if **loss of either *rab-3* and *rbf-1* enhance neuronal Rab-27 axon regeneration** phenotype.

**Response:** The question of whether *rab-27*'s regeneration function in neurons is mechanistically distinct from its function in intestine is very interesting. Here, we show that *rab-27,* together with a well characterized group of genes involved in intestinal secretion, inhibit regeneration. Several of these genes, like *aex-4* and *nlp-40*, are expressed only in intestine, and we show that intestinal expression of *rab-27* is sufficient to fully rescue the *rab-27* mutant phenotype. By contrast, other genes that interact with *rab-27's* regeneration function, like *rab-3,* are expressed only in neurons. Together, these data suggest that the neuronal mechanism of *rab-27* function must be substantially different from the intestine. However, since we do not know the neuronal mechanism, we cannot test this idea directly yet. We added a discussion of this issue to the Discussion on pp. 5-6. We now say:

“ As it does in diverse tissues across species, RAB-27 also regulates the tethering and fusion of non-synaptic vesicles in *C. elegans* neurons (23), and as with the intestine, neuronal RAB-27 may regulate the secretion of an unknown ligand or ligands through dense core vesicles to inhibit regeneration. However, important differences underlie the potential inhibitory mechanisms of neuronal and intestinal RAB-27-dependent regeneration inhibition. As we have shown, intestinal RAB-27 mediates regeneration inhibition in part through a specific pathway that regulates homeostatic intestine-to-neuron communication, and relies on several components, such as SNAP25/*aex-4* and *nlp-40* that are exclusively expressed in the *C. elegans* intestine (11). Several possibilities could explain neuronal RAB-27’s incomplete rescue of high regeneration compared to intestinal RAB-27: the two tissue-specific RAB-27-dependent pathways may be regulating the release of different inhibitory ligands or ligand cohorts, with the intestine secreting the more potent inhibitor(s). Alternatively, intestinal and neuronal RAB-27 could regulate the release of the same inhibitory signals, but through distinct secretory pathways of different effectiveness."

3) rab-3 and rbf-1 do not have roles in axon regeneration, but loss of either can enhance intestinal Rab-27 axon defect. Has the author tested if loss of either rab-3 and rbf-1 enhance neuronal Rab-27 axon regeneration phenotype?

**Response:** We agree that experiments focusing on the neuronal pathway would want to test the interaction of neuronal genes such as *rab-3* and many others with the neuronal function of *rab-27*. However, this work focuses on the intestinal pathway.

3) The author rules out genes like *hid-1* as not having roles in axon regeneration based off no phenotype associated with axon regeneration, but does not generate the **double mutant to fully determine if loss of function in *hid-1*** would act like *rab-3* or *rbf-1*, either enhance or suppress Rab-27 axon regeneration phenotype.

**Response:** Since *hid-1* mutants do not have any regeneration phenotype, we think that concluding it has no critical role in regeneration is supported by the data.

4) The authors **need a working model or schematic** to show the potential genetic interactions for *rab-27* and cellular sites of action in GABA axon regeneration. There are at least two independent regulation pathways for inhibition of axon regeneration, a potential pathway mediated by intestinal *rab-27* and another that may be regulated by neuronal *rab-27*.

**Response:** We appreciate the suggestion, and we now provide a schematic in Fig. 5C, described in the Discussion on p. 5.

6)  Overall, there are places in the manuscript where there could be **more details explaining the rationale** for why the authors are testing particular genes or pathways. In addition, there could be **more details in the conclusions in explaining the results**.

**Response:** We have revised the manuscript at various points as suggested.

**Minor Comments**

1) For fecundity assays what was the temperature animals kept?

**Response:** Worms were grown at 20°C for the duration of the experiment. This information has been added to the materials and methods section.

2) For statistics and graphing of data, what graphing software was used? This information should be provided.

**Response:** Statistics were calculated and graphs were generated using Prism 9.2.0 for MacOS. This information has been added to the materials and methods section.

3) For the figures, the spacing between figures in panel needs to be adjusted. For example, Figure 1A, is too close to Figure 1C legend. The current layout leads to the reader thinking that the control and rab-27 mutant legend is part of panel A.

**Response:** Spacing between datasets in Figure 1 and other figures have been corrected for clarity

4) In many of the graphs, the white bar is covered or missing, make this stand out more.

**Response:** Regeneration graphs have been adjusted to better show white bar representing median. In strains where median regeneration >50%, median bar may remain obscured by individual data points. This will be addressed in figure legends where it occurs.

5) For the bar graphs, the individual data points for each animal tested is not provided. For consistency, show the individual data points.

**Response:** In datasets presented as bar graphs, data are grouped in to Yes/No categories (e.g. full regeneration or not), rather than measurements of individual axons. Sample sizes for each bar are included in the figure legends.

**Reviewer 2:**

**Reviewer 2 commented that "[this]** is an interesting finding, but there are several questions should be carefully addressed to reach the conclusion."

We appreciate the suggestions and have performed additional experiments. We address the specific points in detail below.

1.  In a previous work, authors reported that rab-27 cell-autonomously inhibited axon regeneration. In this study, new evidence suggested that rab-27 might function in both intestine and GABAergic neurons to inhibit axon regeneration. This conclusion was reached by rescue experiments with different promoters and 3’utr. As discussed by the authors, there are many technique limitations for the rescue experiments using multiple copies transgenes. As this is the main conclusion of the manuscript, and it is somewhat inconsistent with the results from their published study, **it is necessary to specifically knock out rab-27 in intestine and neurons** (using Cre-Loxp or FLP-FRT system) to confirm its role in axon regeneration.

**Response:** We attempted to do this experiment using the ZIF-1 degron system (DOI: [10.1242/dev.115048](https://doi.org/10.1242/dev.115048)), which we have found to be very robust for tissue-specific knockdown. We used CRISPR to insert the ZF1 tag and GFP into the endogenous *rab-27* locus, tagging the protein at its N-terminal. Insertion of the ZF1::GFP tag was confirmed by sequencing and other approaches. However, GFP expression was not observed in any tissue. In particular, we were unable to detect GFP in the in the intestine or in the nervous system, even in the synapse-rich area of the nerve ring. Further, these animals were constipated, consistent with loss of *rab-27* function. Expression of ZIF-1 in the intestine had no effect in tagged or control animals (Fig. A). We conclude that the tagged protein is non-functional, and (due to the lack of GFP signal) very likely is misfolded and degraded. These data are quite surprising, since N-terminal tags are routinely used on overexpressed Rab proteins, including RAB-27 in this study.

As an alternative, we performed additional tissue-specific experiments. We now show that re-expression of *nlp-40*, *cab-1*/NPDC1, *aex-4*/SNAP25, and *aex-5*/KPC3 in the intestine is sufficient to restore axon regeneration to normal levels. These experiments confirm the central idea of the manuscript--that components of a well-characterized secretory pathway in the intestine act to inhibit axon regeneration. The new data are described on pages 3, 4 and 5 and shown in Figures 3B, 4B and 5B.

With respect to our previous study, we also provide experiments that clarify the relationship between our functional expression data for *rab-27* in intestine and neurons (Sup. Fig. 1).

Finally, we note that a number of the genes we analyze for their effect on axon regeneration (unlike *rab-27*), are only expressed in the intestine: *aex-4* and *nlp-40*. Together, these data support a role for an intestinal secretion pathway in limiting axon regeneration.

2. NLP-40 was identified as one of the peptides involved in regulating axon regeneration. As in the literature cited by authors, NLP-40 is expressed not only in intestine but also in coelomocytes (potentially others). Therefore, **intestine specific recue of nlp-40(lf) is needed** to reach the conclusion that nlp-40 is released from the intestine to affect axon regeneration. Similarly, **intestine specific rescue of NPDC1/cab-1, SNAP25/aex-4, KPC3/aex-5 are also necessary to reach the conclusion**.

**Response:** We appreciate the suggestion and performed these experiments. Please see #1 above.

3. Also there wasn’t any evidence to show that RAB-27 inhibits axonal regeneration through NLP-40 secretion. To reach this conclusion, the authors at least need to carry out **double mutant analyses of rab-27;nlp-40**

**Response**: We performed additional double mutant analysis. Given the very strong phenotype of *rab-27* mutants, we performed 12 hour regeneration assays to assess *rab-27* double mutants for *cab-1* and *aex-4*, with the idea that this assay would have increased dynamic range. We now show that regeneration in these double mutants is similar to the single mutants. These data are consistent with our proposal that *rab-27, cab-1,* and *aex-4* act in the same pathway to inhibit axon regeneration. Genetic interaction between *rab-27* and *aex-4* suggests a relationship between RAB-27 and NLP-40 secretion, as *aex-4* has previously been shown to be required for NLP-40 secretion (Wang et al. 2013, DOI: [10.1016/j.cub.2013.03.049](https://doi.org/10.1016/j.cub.2013.03.049)). These new data are described on p. 3 and presented in Fig. 4C.

However, regeneration is this time point is ongoing (see 10.1016/j.celrep.2014.06.008) making this assay extremely noisy--as can be seen from the distributions in Fig. 4C. Thus, for mutants with a comparatively weak phenotype (such as *nlp-40*), this approach is extremely difficult to power adequately. In other words, it would be very difficult to be sure about whether or not loss of *nlp-40* increases the already high regeneration phenotype of *rab-27* mutants.

We modified the manuscript to make clear that NLP-40 is only one candidate cargo of the secretory pathway we describe for limiting axon regeneration. We now say (p. 5):

“The strong improvement of regeneration seen in *rab-27, aex-4,* and *cab-1* mutants raises the possibility that these genes may be involved in the secretion of multiple inhibitory signals from the intestine, of which NLP-40 is only one candidate (Fig. 5C). The regeneration phenotypes seen in *nlp-40* and KPC3/*aex-5* mutants, relatively weak compared to those of *cab-1, rab-27* and *aex-4*, suggest that NLP-40 processing and secretion may only represent a part of the inhibitory cargo secreted by this pathway. Identification of additional inhibitory ligands from the intestine will rely on a more complete understanding of the diversity of intestinal vesicles whose secretion is regulated by CAB-1, RAB-27 and AEX-4.”

4. label nlp-40 with GFP (or other tag) and show that axotomy can trigger the release of nlp-40 in a rab-27 dependent manner.

**Response:** We think that the intestinal signaling pathway we characterize here is not triggered by injury, but rather is constitutive. Previous work has demonstrated that NLP-40 secretion from the intestine is constitutive and dependent on *aex-4* (Wang et al. 2013 DOI: [10.1016/j.cub.2013.03.049](https://doi.org/10.1016/j.cub.2013.03.049)). We added a discussion of this point to the Discussion. We now say (p. 5):

“These data suggest a model in which axon regeneration is regulated through secretion of inhibitory ligands from the intestine. This mechanism of regeneration is shared in part with the vesicle secretion and neuropeptide release pathway used for regulation of the defecation motor program (10,13) (Fig. 5C). Because the DMP pathway is constitutively active in adult animals, and NLP-40 in particular is constitutively secreted from the intestine (13), the pathway we describe of intestinal regeneration inhibition is likely not specifically triggered by axon injury, but is more likely incidental to the primary function of this pathway.”

**Minor Comments:**

1. Authors should check the data carefully. n number indicated in the figure legend are inconsistent with the dots number in some figures (for example in figure 1B).

**Response:** Figures have been checked and corrected for inconsistencies including incorrect n.

2. n numbers are very different in each group. How is the n number determined in each group?

**Response:** Axotomy datasets are variable in the number of axons cut due to variability in axotomy effectiveness and worm survival, but measures are taken to ensure consistent representation of data. A minimum of 20 axons was required for each genotype to permit a statistically meaningful assessment of regeneration. Samples with larger datasets reflect either continued analysis throughout the course of the work, or a pooled dataset from multiple experiments involving a particular genotype – in no cases were sample sizes increased to satisfy a statistical conclusion, nor do any larger datasets affect different statistical conclusions than initial n=20 studies.

3. Why the difference of distribution probability of “relative axon length” from the same experiment group in different Figures are largely different? One example is the control group in figure 1B and in figure 4B.

**Response:** Because axon regeneration is a fundamentally variable process, differences in regeneration success can be reflected in the final data. A control regeneration median of 0.6-0.7 after 24 hours of recovery was required for all collected samples, and only these data are included in subsequent analysis. For regeneration analysis at 12 hours, which includes Fig. 1B and Fig. 4B, when regeneration is ongoing (see 10.1016/j.celrep.2014.06.008), variability is even higher than at 24h post-axotomy, which is reflected in the resultant data. It is also important to note that the control genotypes for Fig. 1B and Fig. 4B are different (oxIs12 and juIs76, respectively), and while these two strains do show normal levels of regeneration overall, the intermediate stages of regeneration captured at 12h may be different. In both Fig. 1B and Fig. 4B, axotomy data from mutant lines is only compared to pertinent controls.

4. please label the mean value in an easily distinguishable color.

**Response:** axotomy datasets highlight the median regeneration value as a white bar, which has been adjusted to better show white bar representing median. In strains where median regeneration >50%, median bar may remain obscured by individual data points. This is now addressed in figure legends where it occurs.

**Reviewer 3:**

**Reviewer 3 noted that "** In this manuscript they convincingly demonstrate that Rab-27 functions in the intestine to inhibit regeneration, together with other neuropeptide secretion machinery."

However, it was also noted that " While the core of the paper is reasonably well-supported, the variability of the regeneration assay means that negative results are often over-interpreted and there are some general concerns about reproducibility."

We have addressed these issues and other specific points below.

1. The confirmatory experiments to show that the neuropeptide vesicle release pathway functions in intestinal cells are all done using global mutants. While the double mutant analysis suggests that intestinal function is likely, it would be cleaner to **do some cell-specific manipulations in the intestine**.

**Response**: We appreciate the suggestion and we performed additional tissue-specific experiments. We now show that for *nlp-40*, *cab-1*/NPDC1, *aex-4*/SNAP25, and *aex-5*/KPC3, rescue in the intestine is sufficient to restore axon regeneration to normal levels. These experiments confirm the central idea of the manuscript--that components of a well-characterized secretory pathway in the intestine act to inhibit axon regeneration. The new data are described on pages 3, 4 and 5 and shown in Figures 3B, 4B and 5B.

2. The Munc13b experiment is confusing- mutants are described as blocking secretion involved in the DMP motor program, but no effect was found here. Did the authors check to make sure that DMP is blocked in this genetic background? Similarly, were DMP-related phenotypes checked in hid-1 mutants as a control? And are they really convinced that these are negative results in terms of regeneration? In addition, the regeneration assay seems so variable that I think **a little more caution may be warranted in the interpretation of these negative results** (see below as well).

**Response:** All these *aex-1* and *hid-1* animals were constipated, indicating DMP disruption. We appreciate the point that, due to the variability of axon regeneration, we likely lack the power to have a high chance of detecting subtle phenotypes. We have modified the manuscript to allow for this possibility. We now state (p. 5):

“we do not find a regeneration phenotype for Munc-13b/*aex-1* (Fig. 3A). Munc13 proteins are involved in SNARE-mediated vesicle docking and fusion (37,38), and Munc13-b/*aex-1* loss disrupts intestinal DCV fusion and leads to significant DMP defects (38)… Coregulation of DCV fusion between AEX-1 and other factors could also conceal involvement of this important DCV regulator in regeneration. Alternatively, we may have failed to detect a subtle regeneration effect in *aex-1* mutants.

3. Is it the expulsion motor program that is required to inhibit regeneration? These neurons normally respond to signals from the intestine, so absence of signaling before injury may put these cells into a different program because they are not receiving correct input. Is there another way to block the circuit without disrupting peptide secretion to test whether it is really the secretion per se that is inhibiting regeneration, or whether an inappropriately functioning circuit puts the cells in a pro-regenerative state before injury? Perhaps the aex-2 experiment addresses this? Is circuit function similarly compromised in this mutant background as in the others that improve regeneration? However, I am not convinced that aex-1 and aex-2 (Figure 3) do not improve regeneration. Their average is similar to aex-5, and just because they are not statistically significantly different from controls does not mean there is no effect. It can also mean that not enough animals were tested to detect an effect – which could well be the case because the assay is so variable.

**Response:** The neurons we characterize here--the DD and VD GABA motor neurons--are inhibitory motor neurons that are not involved in the DMP, and are not known to respond to signals from the intestine. The key point of this work is that these motor neurons are in fact responding to intestinal signals--and that these signals inhibit axon regeneration. We show that there is substantial overlap between the intestinal mechanisms that regulate regeneration of VD and DD neurons, and intestinal mechanisms that regulate the DMP in AVD and DVB neurons. It is presently unknown whether AVL, DVB, or other neuron types also respond to the intestinal signals to limit regeneration. In the future, we hope to investigate the neuronal mechanisms that respond to the intestinal signal, and determine in which neuron types they are functional.

With respect to negative data, please see response to point #2.

4. How close in space are the intestine and GABA motor neurons? Is it possible to use existing EMs to show the spatial arrangement of these two cells types relative to one another?

**Response:** EM analysis (i.e. at WormAtlas) shows that the intestine and the VD and DD GABA motor neurons are separated by the pseudocoelum, the fluid-filled body cavity. We added a cartoon to Fig. 5 to make these relationships clear.

5. Intestinal cells normally signal to GABA neurons to control defecation. I am therefore curious whether the influence of intestinal secretion on regeneration is specific to these neurons or acts more generally in cells that are not normally responding to the intestine.

**Response:** Please see response to point #3.

6. Methods seem incomplete. No mention of when axon regeneration was scored, and several timepoints were used. Also, the data acquisition for Figure 2D is not described. And some of the methods seem to be in the figure legends beyond normal amounts- for example the specifics of injection plasmid concentration is in the legend to 2A.

**Response**: Methods have been expanded to better explain axotomy and regeneration scoring strategy, and to include explanation of different timepoints. Analysis of defecation motor program defects has been added, which includes methodology for data in Fig. 2, Fig. S2 and Fig. S3.

**Minor points.**

1. In figure 3 why are some comparisons to oxls12 and some to juls76?

**Response:** Mutant datasets are compared to oxIs12 (*rab-27* and *aex-5*) or juIs76 (all other mutant datasets) based on which GABA::GFP strain was used to visualize axons. Generally, mutants in genes on Chr. X were crossed to juIs76 (integrated on Chr. II), while mutants in genes on the autosomes were crossed to oxIs12 (integrated on Chr. X).

2. Sometimes controls are labeled control, sometimes oxls12. Are they all the same genotype? What is the actual genotype and why was it chosen? What are the different control datasets in different figures? They are quite variable with average axon length at 24h ranging from 0.5ish to 0.8. As some of the phenotypes are quite subtle against the backdrop of this intrinsic variability I have some overall concerns about reproducibility of the data. For example in Figure S3 the control data set seems to have much higher regeneration than other controls, and the aex-1 looks like it regenerates as well as many of the things that are called as having a phenotype in other figures.

**Response:** All mutant lines selected for this study were of confirmed loss-of-function mutants with secondary phenotypes indicative of functional loss – for example, all mutants related to intestinal DCV fusion and neuropeptide secretion were confirmed to show a baseline *aex* phenotype in addition to genotypic mutant confirmation.

The different control datasets in each figure are of control animals cut in parallel to each cohort of mutant lines tested. We have several strategies to control for the inherent variability in axon regeneration, including the use of multiple axotomy sessions per genotype, as well as cutting mutant lines alongside controls, and only comparing regeneration of mutant lines to these specific controls. Minimum sample sizes are also used in each dataset before making any statistical conclusions, and these conclusions are not adjusted or supplemented in datasets with larger sample sizes. This information has been added to the relevant materials and methods section.

3. Rabphilin mis-spelled in one place (as Rabhilin)

**Response:** this and other typos have been corrected in the text and figures

Why is the y axis labeled relative axon length in some figures and normalized axon length in others?

**Response:** this was an error of figure labeling, and has been corrected. All regeneration violin plots should have correctly labeled y-axis as “relative axon length”
